# Supplementary material for: Alteration of Bacterial Communities in Anterior Nares and Skin Sites of Patients Undergoing Arthroplasty Surgery: Analysis by 16S rRNA and Staphylococcal-Specific tuf Gene Sequencing
Source: Microorganisms. 2020 Dec 12;8(12):1977. doi: 10.3390/microorganisms8121977 (PMC7763315; doi:10.3390/microorganisms8121977)
Supplement: Supplementary file 1 [file microorganisms-08-01977-s001.zip › Supplementary/Suppl. tables/Supplementary Table S2.docx]

**Table S2.** Pearson’s product-moment correlation for different *Staphylococcus spp.* between *tuf* gene sequencing and total staphylococcal percentage point change in 16S rRNA (V3-V4) gene sequencing from anterior nares (top) and groin sites (bottom).

| **Staphylococcal Species** | **Estimate** | **P-value** | **Conf.low** | **Conf. high** | **BH adjusted p-value** |
| --- | --- | --- | --- | --- | --- |
| ***S. aureus*** | **0.3660397** | **0.0082513** | **0.1006045** | **0.5828306** | **0.0412563** |
| *S. capitis* | -0.3945410 | 0.0041707 | -0.6044080 | -0.1334694 | 0.0412563 |
| *S. epidermidis* | -0.3119428 | 0.0258526 | -0.5410172 | -0.0397788 | 0.0677400 |
| *S. haemolyticus* | -0.0790146 | 0.5815302 | -0.3470414 | 0.2009447 | 0.7845090 |
| *S. hominis* | -0.0627263 | 0.6619077 | -0.3325612 | 0.2166016 | 0.7845090 |
| *S. lugdunensis* | -0.1667182 | 0.2422848 | -0.4228731 | 0.1141081 | 0.4845696 |
| *S. pasteuri* | -0.0541152 | 0.7060581 | -0.3248542 | 0.2248213 | 0.7845090 |
| *S. saprophyticus* | 0.1415481 | 0.3217854 | -0.1394763 | 0.4014711 | 0.5363089 |
| *S. simulans* | -0.0287743 | 0.8411392 | -0.3019635 | 0.2487822 | 0.8411392 |
| *S. warneri* | 0.3095104 | 0.0270960 | 0.0370904 | 0.5391101 | 0.0677400 |
| *S. aureus* | -0.0186755 | 0.9248520 | -0.3890418 | 0.3568881 | 0.9966235 |
| *S. capitis* | 0.0008380 | 0.9966235 | -0.3723554 | 0.3737980 | 0.9966235 |
| *S. caprae* | -0.0451118 | 0.8196916 | -0.4112670 | 0.3335793 | 0.9966235 |
| *S. epidermidis* | -0.1280179 | 0.5162077 | -0.4782532 | 0.2573502 | 0.9966235 |
| *S. haemolyticus* | 0.3515948 | 0.0665470 | -0.0247255 | 0.6406380 | 0.3534911 |
| *S. hominis* | 0.0708968 | 0.7199703 | -0.3103899 | 0.4325333 | 0.9966235 |
| *S. lugdunensis* | -0.1961499 | 0.3171325 | -0.5304118 | 0.1908966 | 0.7928312 |
| *S. saprophyticus* | 0.3466926 | 0.0706982 | -0.0303039 | 0.6373348 | 0.3534911 |
| *S. sciuri* | -0.2936530 | 0.1293461 | -0.6008984 | 0.0891958 | 0.4311535 |
| *S. simulans* | -0.0625434 | 0.7518737 | -0.4256876 | 0.3179524 | 0.9966235 |
