# Supplementary material for: Alteration of Bacterial Communities in Anterior Nares and Skin Sites of Patients Undergoing Arthroplasty Surgery: Analysis by 16S rRNA and Staphylococcal-Specific tuf Gene Sequencing
Source: Microorganisms. 2020 Dec 12;8(12):1977. doi: 10.3390/microorganisms8121977 (PMC7763315; doi:10.3390/microorganisms8121977)
Supplement: Supplementary file 1 [file microorganisms-08-01977-s001.zip › Supplementary/Suppl. tables/Supplementary Table S3.docx]

**Table S3.** Primer table of *tuf* gene sequencing primers with heterogeneity spacers and Illumina adaptors as used in this study.

| Forward  Primers | Forward Illumina adaptors  5’ | Primer sequence with **heterogeneity spacers** |
| --- | --- | --- |
| Tuf_F01 | TCGTCGGCAGCGTCAGATGTGTATAAGAGACAG | CAGAAGAAAAAGAACGTGG |
| Tuf_F02 | TCGTCGGCAGCGTCAGATGTGTATAAGAGACAG | **T**CAGAAGAAAAAGAACGTGG |
| Tuf_F03 | TCGTCGGCAGCGTCAGATGTGTATAAGAGACAG | **GT**CAGAAGAAAAAGAACGTGG |
| Tuf_F04 | TCGTCGGCAGCGTCAGATGTGTATAAGAGACAG | **AGTCTCTCGT**CAGAAGAAAAAGAACGTGG |
| Reverse  Primers | Reverse Illumina adaptors  5’ | Primer sequence with **heterogeneity spacers** |
| Tuf_R01 | GTCTCGTGGGCTCGGAGATGTGTATAAGAGACAG | GTCCTCAACWGGCATCA |
| Tuf_R02 | GTCTCGTGGGCTCGGAGATGTGTATAAGAGACAG | **A**GTCCTCAACWGGCATCA |
| Tuf_R03 | GTCTCGTGGGCTCGGAGATGTGTATAAGAGACAG | **CA**GTCCTCAACWGGCATCA |
| Tuf_R04 | GTCTCGTGGGCTCGGAGATGTGTATAAGAGACAG | **TCAG**GTCCTCAACWGGCATCA |
