# Supplementary material for: Alteration of Bacterial Communities in Anterior Nares and Skin Sites of Patients Undergoing Arthroplasty Surgery: Analysis by 16S rRNA and Staphylococcal-Specific tuf Gene Sequencing
Source: Microorganisms. 2020 Dec 12;8(12):1977. doi: 10.3390/microorganisms8121977 (PMC7763315; doi:10.3390/microorganisms8121977)
Supplement: Supplementary file 1 [file microorganisms-08-01977-s001.zip › Supplementary/Suppl. tables/Supplementary Table S1.docx]

**Table S1.** Overview of samples and read counts for 16S rRNA (V3-V4) and *tuf* gene sequencing.

|  | **Nares** | **Groin** | **Operation site** |
| --- | --- | --- | --- |
| **Patients sampled before and after arthroplasty** | **79** | **61** | **37** |
| Median age (range) | 68 (45-83) | 70 (45-83) | 69 (50-83) |
| Female  Male | 44 (56%)  36 (44%) | 35 (57%)  26 (43%) | 22 (59%)  15 (41%) |
| **16S (V3-V4) Gene Sequencing Data** |  |  |  |
| Average raw sequencing read counts | 18,015 | 17,376 | 10,076 |
| Average read counts after *decontam()* | 14,171 | 13,127 | 3,429 |
| Patients with >2,000 reads in samples before surgery | 65 (82%)^1^ | 54 (89%)^1^ | 13 (35%)^1^ |
| Patients with >2,000 reads in samples after surgery | 65 (82%)^1^ | 42 (69%)^1^ | 7 (19%)^1^ |
| Patients with >2,000 reads for both time points | 59 (75%)^1^ | 39 (64%)^1^ | 4 (11%)^1^ |
| Average read count in samples used for analysis | 17,127 | 17,887 | 10,678 |
| ***Tuf* Gene Sequencing Data** |  |  |  |
| Average raw sequencing read counts | 38,653 | 31,334 | 20,805 |
| Average reads mapped to the *tuf* gene database | 38,416 | 30,820 | 20,328 |
| Patients with >2,000 reads in samples before surgery | 71 (90%)^1^ | 58 (95%)^1^ | 32 (87%)^1^ |
| Patients with >2,000 reads in samples after surgery | 72 (91%)^1^ | 47 (77%)^1^ | 23 (62%)^1^ |
| Patients with >2,000 reads for both time points | 65 (82%)^1^ | 41 (67%)^1^ | 20 (54%)^1^ |
| Average read count in samples used for analysis | 42,107 | 35,029 | 27,114 |

^1^ Percentages indicate communities remaining for each sampling site after filtering samples with <2000 read counts.
