# Supplementary material for: Alteration of Bacterial Communities in Anterior Nares and Skin Sites of Patients Undergoing Arthroplasty Surgery: Analysis by 16S rRNA and Staphylococcal-Specific tuf Gene Sequencing
Source: Microorganisms. 2020 Dec 12;8(12):1977. doi: 10.3390/microorganisms8121977 (PMC7763315; doi:10.3390/microorganisms8121977)
Supplement: Supplementary file 1 [file microorganisms-08-01977-s001.zip › Supplementary/Suppl. figures/Supplementary Figure S6.docx]

**Figure S6.** Prevalence of staphylococcal species according to sampling site before and after surgery (A) without a lower cut-off and (B) with a cut-off keeping only staphylococcal ASVs ≥1% of total *tuf* sequencing reads in each sample.
