# Supplementary material for: Alteration of Bacterial Communities in Anterior Nares and Skin Sites of Patients Undergoing Arthroplasty Surgery: Analysis by 16S rRNA and Staphylococcal-Specific tuf Gene Sequencing
Source: Microorganisms. 2020 Dec 12;8(12):1977. doi: 10.3390/microorganisms8121977 (PMC7763315; doi:10.3390/microorganisms8121977)
Supplement: Supplementary file 1 [file microorganisms-08-01977-s001.zip › Supplementary/Suppl. figures/Supplementary Figure S7.docx]

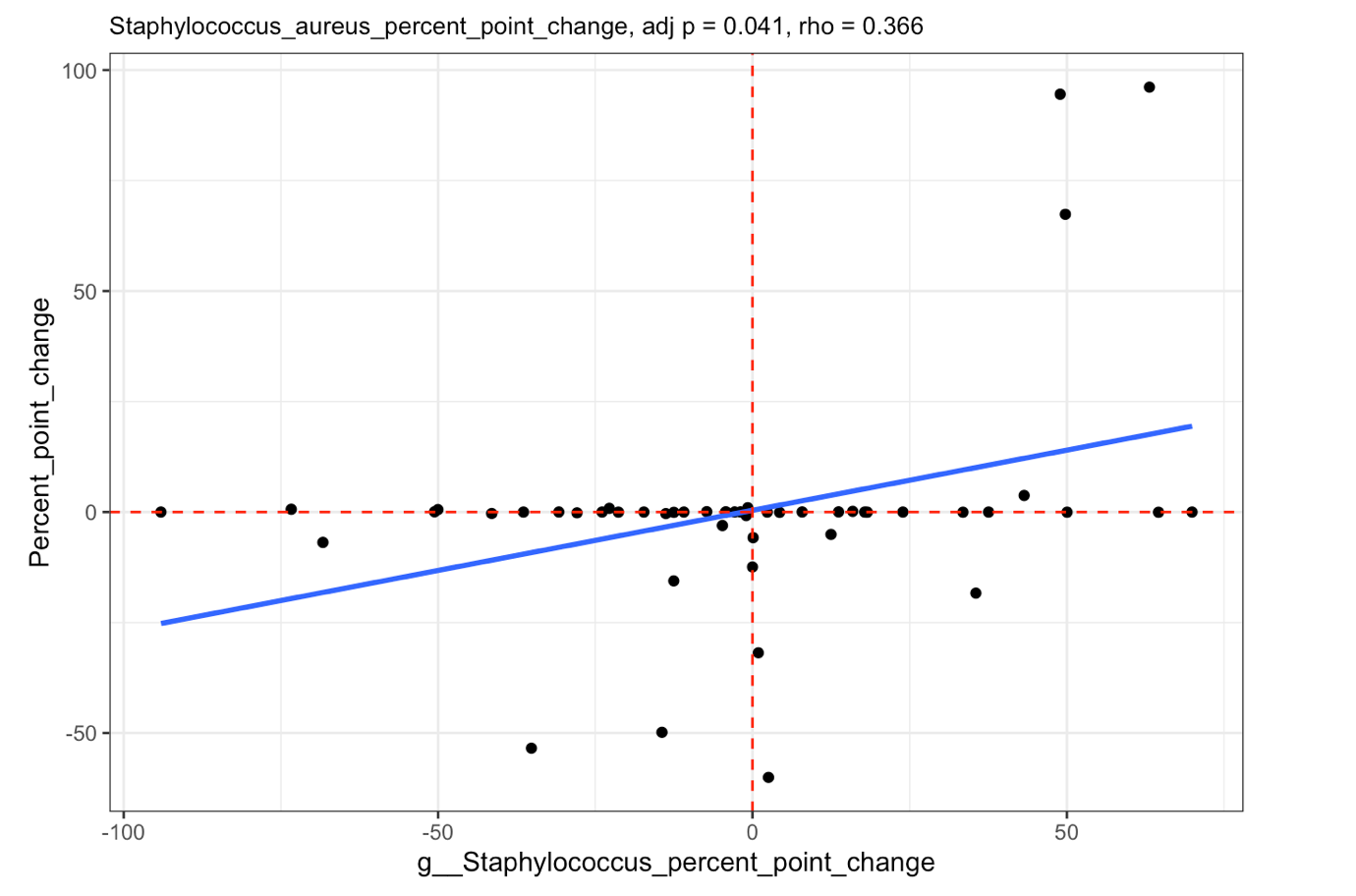


**Figure S7.** Correlation between alterations in *S. aureus* ASVs in *tuf* gene sequencing and total staphylococcal percentage point change in 16S rRNA (V3-V4) gene sequencing in nares of patients before compared to after arthroplasty surgery.
