# Supplementary material for: Alteration of Bacterial Communities in Anterior Nares and Skin Sites of Patients Undergoing Arthroplasty Surgery: Analysis by 16S rRNA and Staphylococcal-Specific tuf Gene Sequencing
Source: Microorganisms. 2020 Dec 12;8(12):1977. doi: 10.3390/microorganisms8121977 (PMC7763315; doi:10.3390/microorganisms8121977)
Supplement: Supplementary file 1 [file microorganisms-08-01977-s001.zip › Supplementary/Suppl. figures/Supplementary Figure S3.docx]

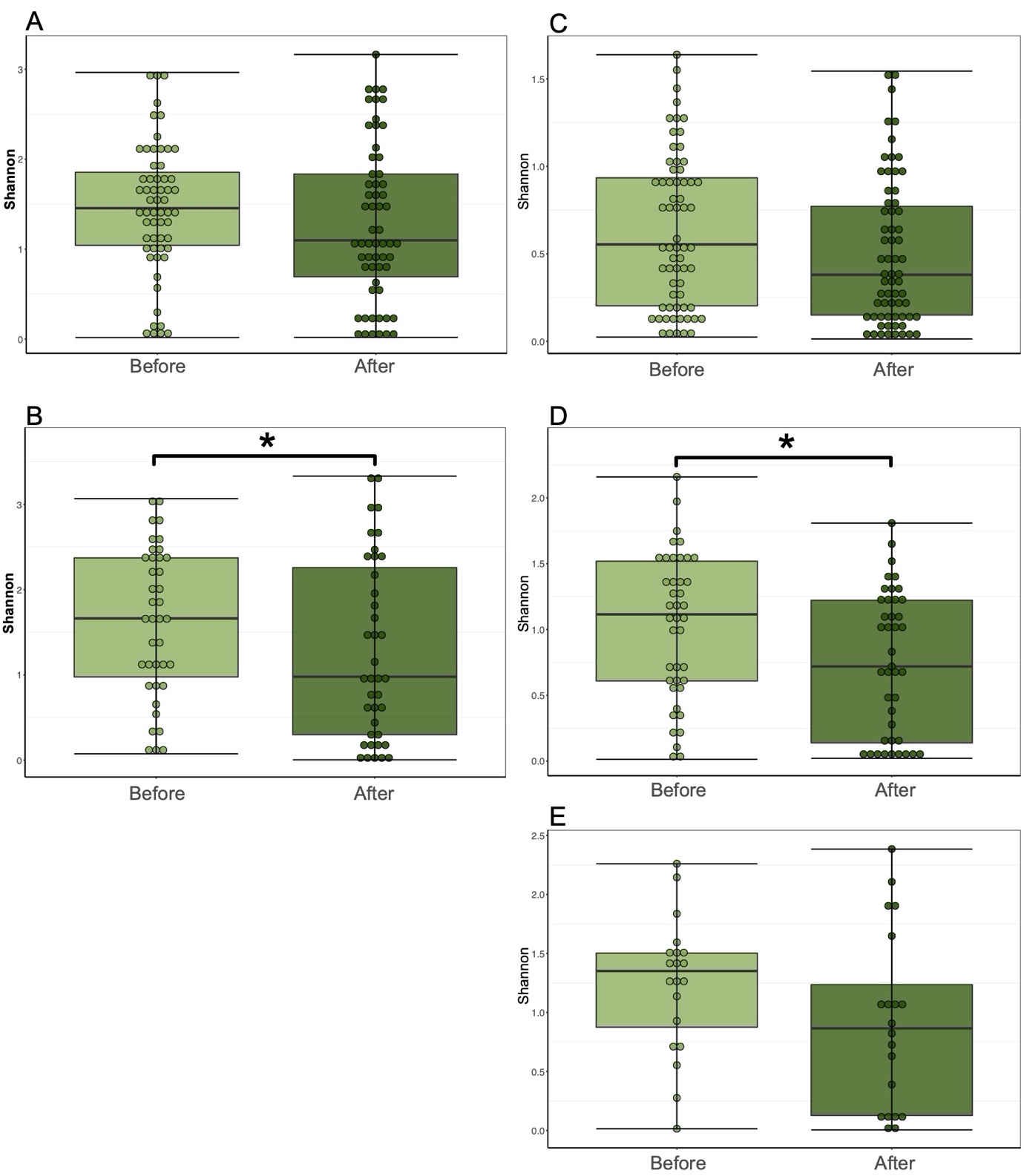


**Figure S3.** Alpha (Shannon) diversity plots for 16S rRNA gene sequencing of (A) anterior nares and (B) groin samples before and after surgery. Alpha (Shannon) diversity plots for *tuf* gene sequencing of (C) anterior nares, (D) groin, and (E) operation sites samples before and after surgery. Asterisks signifies a significant change with a p-value < 0.05.
