# Supplementary material for: Alteration of Bacterial Communities in Anterior Nares and Skin Sites of Patients Undergoing Arthroplasty Surgery: Analysis by 16S rRNA and Staphylococcal-Specific tuf Gene Sequencing
Source: Microorganisms. 2020 Dec 12;8(12):1977. doi: 10.3390/microorganisms8121977 (PMC7763315; doi:10.3390/microorganisms8121977)
Supplement: Supplementary file 1 [file microorganisms-08-01977-s001.zip › Supplementary/Suppl. figures/Supplementary Figure S5.docx]

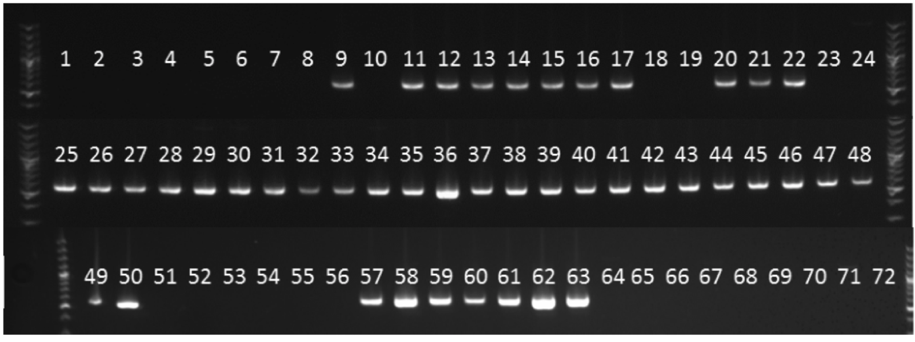


| 1 | *Corynebacterium ulcerans* | 37 | *Staphylococcus schleiferi* |
| --- | --- | --- | --- |
| 2 | *Enterococcus faecalis* | 38 | *Staphylococcus sciuri* |
| 3 | *Enterococcus faecalis* | 39 | *Staphylococcus sciuri* |
| 4 | *Enterococcus faecium* | 40 | *Staphylococcus sciuri* |
| 5 | *Micrococcus luteus* | 41 | *Staphylococcus simulans* |
| 6 | *Micrococcus luteus* | 42 | *Staphylococcus simulans* |
| 7 | *Moraxella bovis* | 43 | *Staphylococcus succinus* |
| 8 | *Chryseobacterium indologenes* | 44 | *Staphylococcus succinus* |
| 9 | *Staphylococcus capitis* | 45 | *Staphylococcus vitulinus* |
| 10 | *Streptococcus spp.* | 46 | *Staphylococcus vitulinus* |
| 11 | *Staphylococcus chromogenes* | 47 | *Staphylococcus warneri* |
| 12 | *Staphylococcus chromogenes* | 48 | *Staphylococcus warneri* |
| 13 | *Staphylococcus cohnii subsp. urealyticus* | 49 | *Staphylococcus xylosus* |
| 14 | *Staphylococcus spp.* | 50 | *Staphylococcus xylosus* |
| 15 | *Staphylococcus spp.* | 51 | *Streptococcus haemolyticus* |
| 16 | *Staphylococcus epidermidis* | 52 | *Streptococcus pneumoniae* |
| 17 | *Staphylococcus epidermidis* | 53 | *Streptococcus pyogenes* |
| 18 | *Streptococcus spp.* | 54 | *Streptococcus sanguis* |
| 19 | *Streptococcus spp.* | 55 | *Streptococcus suis* |
| 20 | *Staphylococcus spp.* | 56 | *Streptococcus salivarious* |
| 21 | *Staphylococcus equorum* | 57 | *Staphylococcus lugdunensis (104016)* |
| 22 | *Staphylococcus felis* | 58 | *Staphylococcus lugdunensis (ATCC 70328)* |
| 23 | *Streptococcus spp.* | 59 | *Staphylococcus schweitzeri* |
| 24 | *Streptococcus spp.* | 60 | *Staphylococcus argenteus* |
| 25 | *Staphylococcus hominis* | 61 | *Staphylococcus cohnii* |
| 26 | *Staphylococcus hyicus* | 62 | *Staphylococcus saprophyticus* |
| 27 | *Staphylococcus hyicus* | 63 | *Staphylococcus intermedius* |
| 28 | *Staphylococcus hyicus subsp. chromogenes* | 64 | *Moraxella nonliquefaciens* |
| 29 | *Staphylococcus pseudintermedius* | 65 | *Moraxella catarrhalis* |
| 30 | *Staphylococcus pseudintermedius* | 66 | *Corynebacterium pseudotuberculosis* |
| 31 | *Staphylococcus lentus* | 67 | *Corynebacterium minutissimum* |
| 32 | *Staphylococcus epidermids* | 68 | *Corynebacterium striatum* |
| 33 | *Staphylococcus pseudintermedius* | 69 | *Corynebacterium jeikeium* |
| 34 | *Staphylococcus pseudintermedius* | 70 | *Corynebacterium xerosis* |
| 35 | *Staphylococcus pulvereri* | 71 | *Corynebacterium urealyticum* |
| 36 | *Staphylococcus schleiferi* | 72 | *Corynebacterium amycolatum* |

**Figure S5.** Agarose gel with *tuf* PCR products from staphylococcal and non-staphylococcal bacteria as outlined in the table.
