# Supplementary material for: Alteration of Bacterial Communities in Anterior Nares and Skin Sites of Patients Undergoing Arthroplasty Surgery: Analysis by 16S rRNA and Staphylococcal-Specific tuf Gene Sequencing
Source: Microorganisms. 2020 Dec 12;8(12):1977. doi: 10.3390/microorganisms8121977 (PMC7763315; doi:10.3390/microorganisms8121977)
Supplement: Supplementary file 1 [file microorganisms-08-01977-s001.zip › Supplementary/Suppl. figures/Supplementary Figure S9.docx]

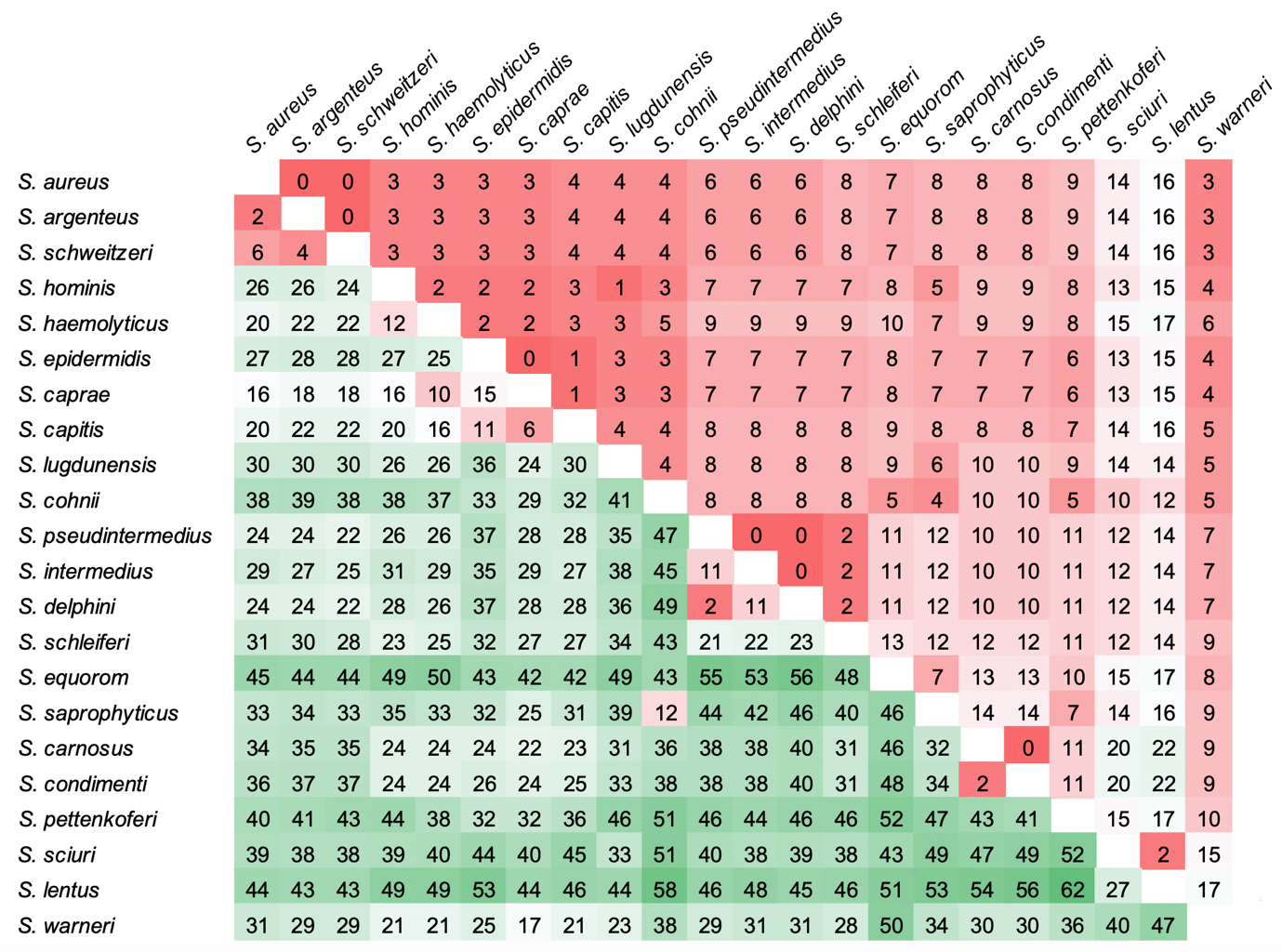


**Figure S9.** Single nucleotide polymorphism (SNP) differences between staphylococcal species in the 16S rRNA (V3-V4) gene region sequenced in this study (top) and the amplified *tuf* gene region (bottom) calculated from the most prevalent ASV from each staphylococcal species.
